# mGlu2 Receptors in the Basal Ganglia: A New Frontier in Addiction Therapy

**DOI:** 10.31083/FBL26637

**Published:** 2025-08-28

**Authors:** Li-Min Mao, Elizabeth Puthumana, John Q. Wang

**Affiliations:** 1 Department of Biomedical Sciences, University of Missouri-Kansas City, School of Medicine, Kansas City, MO 64108, USA; 2 Department of Anesthesiology, University of Missouri-Kansas City, School of Medicine, Kansas City, MO 64108, USA

**Keywords:** glutamic acid, dopamine, autoreceptors, basal ganglia, substance-related disorders, cocaine, amphetamine, nicotine, ethanol, opioid

## Abstract

Glutamate is an important neurotransmitter in the mammalian brain. Among the receptors that glutamate interacts with is metabotropic glutamate (mGlu) receptor 2, a G*α*_i/o_-coupled receptor. These receptors are primarily located on glutamatergic nerve terminals and act as presynaptic autoreceptors to produce feedback inhibition of glutamate release. Abundant mGlu2 receptors are distributed in major glutamatergic pathways in the basal ganglia, especially the corticostriatal and thalamostriatal projections in the striatum. These receptors are involved in the regulation of motivation, reward processing, learning, motor, and cognitive functions. As an inhibitory presynaptic receptor, mGlu2 is linked to the addictive properties of drugs of abuse, a topic summarized in this review. Chronic exposure to multiple addictive drugs and alcohol causes the adaptive downregulation of mGlu2 receptors in their expression and function in the key regions of the limbic reward circuit. This downregulation contributes to the remodeling of limbic excitatory synaptic transmission and plasticity critical for enduring drug-seeking behavior. Normalization of mGlu2 activity by pharmacological or genetic approaches attenuates drug taking and seeking. Here, we highlight that recent progress in mGlu2 biology research demonstrates the pivotal roles of mGlu2 receptors in different aspects of drug addiction. mGlu2 subtype-selective agents (both orthosteric and allosteric compounds) thus have the potential to be developed into novel pharmacotherapies for addictive conditions.

## 1. Introduction

Metabotropic glutamate (mGlu) receptors are G protein-coupled receptors densely expressed in the mammalian brain. Eight mGlu subtypes (mGlu1-8) are subdivided into three functional groups (I–III) based on sequence homology, associated post-receptor signaling transduction pathways, and pharmacological properties [1]. Group II receptors are comprised of mGlu2 and mGlu3 subtypes. Both are coupled to the G*α*_i/o_ heterotrimeric G proteins and show similarities and differences in localization, expression level, and physiology. Activation of the mGlu2 subtype inhibits adenylyl cyclase and thereby reduces cyclic adenosine monophosphate (cAMP) production and protein kinase A (PKA) activity [1]. Additionally, mGlu2 receptors modulate many other cytoplasmic and synaptic effectors, including extracellular signal-regulated kinases (ERK), voltage-gated Ca^2+^ channels (VGCC) and G-protein-coupled inwardly rectifying K^+^ (GIRK) channels, and induce a chemical form of synaptic plasticity, i.e., mGlu2-dependent long-term depression (LTD), at glutamatergic synapses [1,2].

The *GRM2* gene encodes human mGlu2 receptor proteins [mGlu2 receptor accession numbers: NP_000830 (human), NP_001099181 (rat), and NP_001153825 (mouse)] [2-4]. The mGlu2 amino acid sequence shares approximately 70% homology with the mGlu3 receptor [5]. Unlike *GRM3* encoding mGlu3 receptors, no alternative splicing of *GRM2* has been observed at present. As with other mGlu receptors, mGlu2 receptors function primarily in the form of homodimers (mGlu2/2). Additionally, mGlu2 can heterodimerize with mGlu4 to form functional mGlu2/4 heterodimers in brain cells *in vivo* [6,7] and with mGlu7 to form an mGlu2/mGlu7 heterodimer structure in which mGlu7 predominantly controls dimeric association and G-protein activation [8].

mGlu2 receptors are expressed in neurons but not in glial cells. They are enriched at synaptic sites and are predominantly presynaptic, as opposed to group I receptors (mGlu1/5) which are mostly postsynaptic. At the ultrastructural level, mGlu2 receptors like mGlu3 reside in an area outside of the active zone of axon terminals, differing from group III receptors that are localized within the active zone [9]. Such perisynaptic arrangement positions mGlu2 to mainly sense synaptic glutamate overflow and glutamate from astrocytes. Notably, abundant mGlu2 receptors are present on glutamatergic presynaptic nerve terminals in the basal ganglia. These autoreceptors produce robust feedback inhibition of glutamate release and play pivotal roles in various neuropsychiatric disorders, including drugs of abuse.

Substance addiction is a common neuropsychiatric disorder with less clear brain mechanisms underlying its etiology. Accumulative evidence indicates pivotal roles of dysregulated glutamatergic transmission in the pathophysiology of drug-seeking behavior [10], particularly for glutamatergic hyperactivity in the limbic reward circuit. Remarkably, recent preclinical studies in animals found that mGlu2 receptors are sensitive to drugs and may contribute to drug-induced remodeling of glutamatergic transmission. Namely, as summarized in this review, chronic exposure to substances like psychostimulants (e.g., cocaine and amphetamines), nicotine, and alcohol induces long-lasting adaptive downregulation of presynaptic mGlu2 autoreceptors. This adaptive change affects mGlu2 expression and function in the nucleus accumbens (NAc) and other key limbic reward regions. This reduces mGlu2-mediated feedback inhibition of glutamate release, leading to enhanced synaptic glutamatergic transmission, which is critical for persistent drug-seeking behavior. As such, restoration of mGlu2 activity could effectively attenuate the addictive properties of drugs. Novel mGlu2 selective agents (either orthosteric or allosteric compounds) are therefore of therapeutic value for treating addiction [11-13]. This review aims to summarize the literature on the relationship between mGlu2 receptors and drug addiction and to clarify the role of mGlu2 receptors in shaping long-term adaptive changes in limbic glutamatergic transmission related to persistent drug-seeking behavior.

## 2. Distribution of mGlu2 Receptors in the Basal Ganglia

The striatum is the largest structure in the basal ganglia and is divided into the ventral NAc and the dorsal caudate putamen (CPu) [14]. Medium spiny projection neurons (MSN) comprise 95% of the total striatal neuronal population. These *γ*-aminobutyric acid (GABA)ergic neurons are segregated into two major phenotypes: D_1_-bearing striatonigral neurons projecting to the substantia nigra pars reticulata (SNr) and internal globus pallidus (direct pathway) and D_2_-bearing striatopallidal neurons projecting to the external globus pallidus (indirect pathway). In addition to projection neurons, the striatum contains several types of interneurons, including the large aspiny cholinergic interneuron (CIN). The CPu and NAc receive dopaminergic inputs from the substantia nigra pars compacta (SNc) and the ventral tegmental area (VTA), respectively. Other crucial inputs are glutamatergic. In the CPu, these inputs converge from the cortex and thalamus, while in the NAc, they come from the prefrontal cortex (PFC), ventral hippocampus, basolateral amygdala, and thalamus.

*In situ* hybridization studies were carried out to map mGlu2 mRNA expression in the rat brain [15,16]. It was found that mGlu2 mRNAs were specifically labeled in neuronal cells. Neurons in the whole cortex, hippocampus, amygdala, thalamus, and subthalamic nucleus expressed moderate to high levels of mGlu2 mRNAs. Weakly labeled neurons were sparsely scattered in the striatum. No and very low levels of mGlu2 mRNAs were detected in the substantia nigra (SNr and SNc) and VTA, respectively. At the protein level, several immunohistochemical studies used a dual mGlu2/3 polyclonal antibody [17-21]. In immunohistochemical studies with an mGlu2 selective monoclonal antibody [19,22-24], no glial cells were found to express mGlu2 receptors, in contrast to mGlu3 receptors that are expressed in both neurons and glial cells throughout the brain [25]. However, a study found weak mGlu2 expression in astrocytes in the monkey dorsolateral PFC [26]. Of note, neuropil immunostaining of mGlu2 was intense in the CPu and NAc [23], corresponding to robust ligand binding of mGlu2 receptors in these regions [27,28]. The set of mGlu2 receptors labeled in the striatum is assumed to be mainly presynaptic receptors located on axon terminals of glutamatergic corticostriatal and thalamostriatal projection fibers [23]. In support of this, decortication reduced mGlu2/3 binding and immunoreactivity in the striatum, although responses of the individual group II subtypes were not examined [20,29]. Besides mGlu2, mGlu3 receptors are localized in anterogradely-labeled corticostriatal axon terminals that form asymmetric (excitatory) synapses on striatal neurons [24]. Both mGlu2 and mGlu3 immunoreactivity was never associated with GABAergic axon fibers in the striatum [30], tyrosine hydroxylase-containing dopaminergic axon terminals in the striatum, and dopamine neuronal soma in the SNc [20,30]. Unlike abundant neuropil staining, mGlu2 immunoreactive neuronal cell bodies were only distributed sparsely in the striatum [23]. Most of these neurons were rather large and aspiny and were subsequently confirmed to be CINs as mGlu2 with little or no mGlu3 mRNAs were expressed in these biochemically identified CINs [31,32]. In addition to the striatum, weak neuropil labeling of mGlu2 immunoreactivity was seen in the SNc, SNr, VTA, and globus pallidus [23]. At synaptic sites, mGlu2 receptors are predominantly presynaptic, while the receptor could also be postsynaptic, e.g., in Golgi cells in the cerebellar cortex [22]. In the rat hippocampus, mGlu2 receptors reside in an area outside of the active zone of axon terminals as opposed to group III receptors that are located within the active zone [9]. In the monkey dorsolateral PFC, mGlu2 receptors were either targeted to the active zone or localized perisynaptically [26]. A perisynaptic location allows the receptor to preferentially sense glutamate overflow and glutamate from astrocytes [9,10,18].

## 3. mGlu2 Receptor Signaling and Physiology

**Fig. 1.**
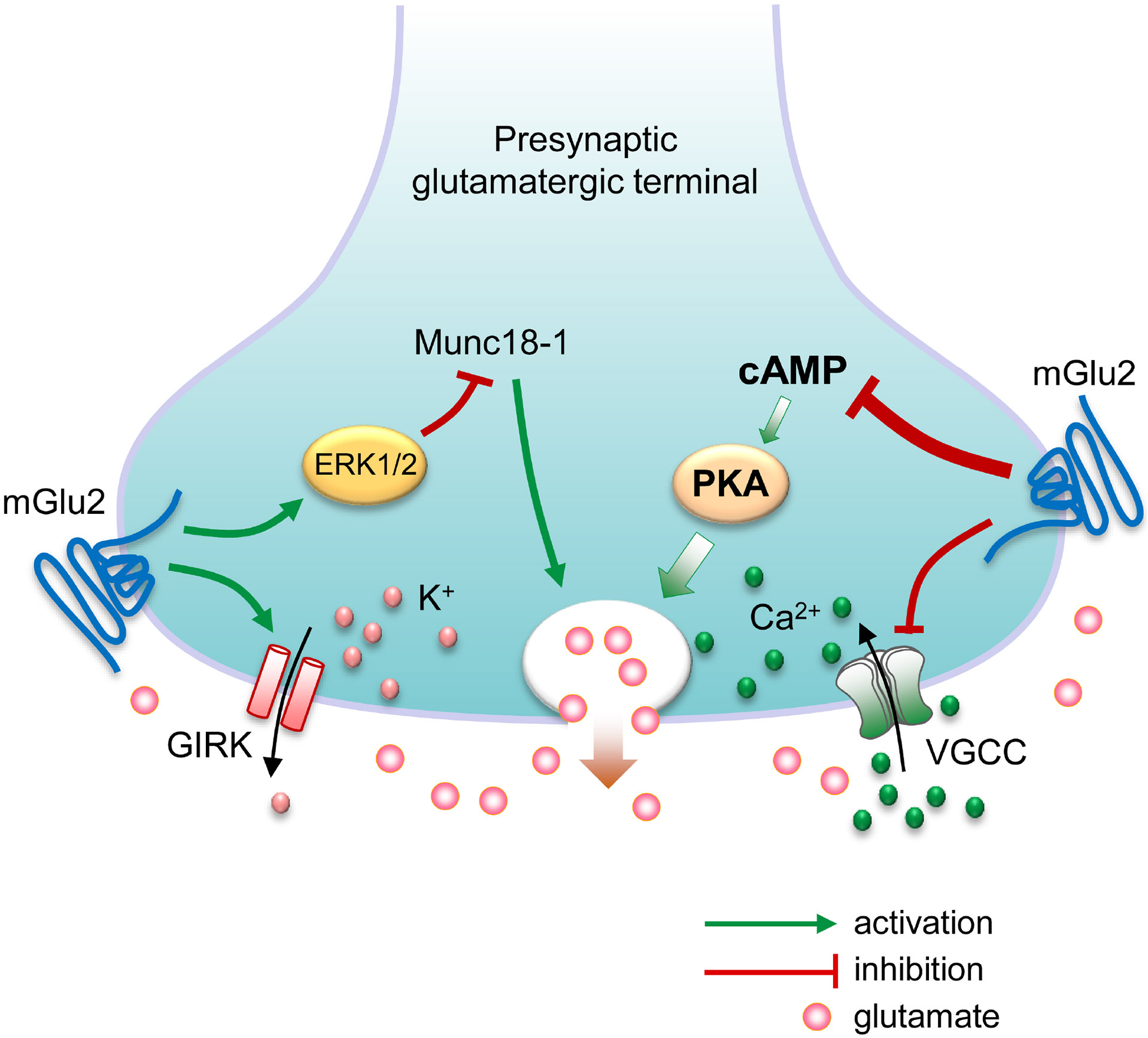
Possible molecular mechanisms underlying presynaptic metabotropic glutamate (mGlu) receptor 2-mediated feedback inhibition of glutamate release. Presynaptic membrane depolarization causes voltage-gated Ca^2+^ channels (VGCC) to open, allowing the influx of Ca^2+^ ions that triggers glutamate to release. On the other hand, activation of G-protein-coupled inwardly rectifying K^+^ (GIRK) channels results in the efflux of K^+^ ions, leading to presynaptic membrane hyperpolarization and reduction of glutamate release. Presynaptic G*α*_i/o_-coupled mGlu2 receptors could reduce glutamate release by inhibiting cyclic adenosine monophosphate (cAMP)-dependent protein kinase A (PKA) activity, inhibiting VGCCs, and/or activating GIRK channels. Additionally, mGlu2 receptors could negatively modulate glutamate release by activating the extracellular signal-regulated kinase 1/2 (ERK1/2). Active ERK1/2 in turn phosphorylate and thereby negatively regulate Munc18-1, a presynaptic protein essential for synaptic vesicle release.

As G*α*_i/o_-coupled receptors, mGlu2 inhibits adenylyl cyclase and as a result, reduces cAMP production and PKA activity [1]. In addition to this canonical signaling pathway, mGlu2 receptors are positively coupled to the ERK1/2 pathway in heterologous cells [33-35] and likely in cultured rat cortical neurons [36]. Active ERK1/2 could subsequently phosphorylate and thereby negatively affect Munc18-1, a presynaptic protein essential for synaptic vesicle exocytosis [37]. mGlu2 receptors also inhibit VGCCs and activate GIRK channels [38,39]. In heterologous cells and cultured mouse cortical neurons, mGlu2 receptors transactivate insulin-like growth factor 1 receptors via a G*βγ* subunits/phospholipase C/focal adhesion kinase pathway, leading to ERK1/2 activation [40]. Each of the above signaling connections, in addition to possible others [41], could contribute to the negative feedback modulation of transmitter release (Fig. 1). Additionally, mGlu2 interacts with the neurotrophin receptor TrkB and triggers phosphorylation of TrkB at tyrosine 816 in the mouse PFC [42]. As with other mGlu receptors, mGlu2 receptors function primarily as homodimers (mGlu2/2) *in vivo*. Recent studies reveal an asymmetric dimerization mechanism crucial for mGlu2 receptor activation [43-45]. Besides, mGlu2 heterodimerizes with (1) mGlu3 to form mGlu2/3 heterodimers that underwent conformational rearrangement upon activation [46,47], (2) mGlu4 to form mGlu2/4 heterodimers in brain cells that represent the most studied pair among heterodimer mGlu subtypes surveyed [6,7,48-53], and (3) mGlu7 to form mGlu2/7 heterodimers in which mGlu7 dominantly controls dimeric association and G-protein activation [8]. Additionally, mGlu2 forms heterodimers with 5-hydroxytryptamine 2A receptors [54-56], which is critical for the functional crosstalk between the two receptors [54].

As aforementioned, striatal activity is driven by glutamatergic inputs to MSNs from the cortex, thalamus, and other subcortical regions. Several electrophysiological and neurochemical studies revealed that a dual mGlu2/3 agonist (e.g., CHPG, DCG-IV, L-CCG-I, LY354740, or LY379268) inhibited evoked Ca^2+^ influx in corticostriatal axon terminals in the striatum, suppressed excitatory corticostriatal transmission, and induced LTD at corticostriatal synapses [30,57-60]. Since the mGlu2/3 agonist increased paired-pulse ratios at corticostriatal synapses, the agonist is believed to act presynaptically to reduce glutamate release probability [59,61]. Other studies used mGlu2 selective agents and mGlu2 knockout mice to determine the subtype-specific role of mGlu2 receptors in regulating corticostriatal and thalamostriatal transmission. Johnson and co-workers [62] found that mGlu2 receptors mediate depression of striatal excitatory transmission broadly evoked by electrical stimulation via a presynaptic mechanism, while mGlu3 receptors are less likely to play a role in this event. More importantly, using optogenetic techniques that distinguish corticostriatal versus thalamostriatal pathways, Johnson *et al*. [62] provide direct evidence that presynaptic mGlu2 receptors similarly depress excitatory transmission at both corticostriatal and thalamostriatal synapses in the dorsal striatum. Further evidence supporting the role of mGlu2 receptors includes (1) the ability of mGlu2 potentiators to inhibit excitatory synaptic responses to stimulation of corticostriatal afferents [63] and (2) a loss of efficacy of an mGlu2/3 agonist in suppressing evoked field potentials in the striatum of mice lacking mGlu2 receptors [64]. In addition to the striatum, other basal ganglia sites show the mGlu2-mediated negative regulation of excitatory synaptic transmission. In the globus pallidus, mGlu2/3 receptors are present on glutamatergic preterminal axons, and the mGlu2 positive allosteric modulator (PAM) LY487379 potentiated the mGlu2/3 agonist-induced inhibition of local excitatory synaptic transmission [65]. In the SNr, activation of mGlu2 rather than mGlu3 receptors induced LTD at glutamatergic subthalamic nucleus-SNr synapses [66].

mGlu2 receptors also regulate basal and drug-evoked dopamine release in the striatum, likely via an indirect mechanism. mGlu2 mRNAs were not detected in the substantia nigra [15]. No mGlu2 receptors reside on dopamine fibers in the striatum and dopamine neurons in the SNc [20,30]. Thus, mGlu2 receptors may not modulate local dopamine release by acting as a heteroreceptor on dopaminergic axon terminals. Indeed, mGlu2/3 activation did not affect striatal dopamine release induced by direct electrical stimulation of dopamine neurons in the midbrain [67]. In contrast, mGlu2/3 activation reduced basal and drug (amphetamine or cocaine)-induced dopamine release in the striatum [67-70]. Mechanisms underlying this negative regulation of dopamine release are not completely understood. An indirect mechanism may play a role [62]. Namely, it is known that both cortical and thalamic glutamatergic inputs drive CINs to release acetylcholine, which in turn activates nicotinic receptors on dopamine terminals to release dopamine in a way that bypasses activity in dopamine neurons [71-73]. Thus, mGlu2 receptors on corticostriatal and/or thalamostriatal terminals could inhibit CINs via presynaptic actions [31] and thereby lower dopamine levels. Alternatively, activation of mGlu2 heteroreceptors on CIN axon terminals could inhibit acetylcholine release, thereby reducing the cholinergic stimulation of dopamine release. A subtype-specific role of mGlu2 receptors in the regulation of the acetylcholine-dopamine interplay is supported by findings that (1) mGlu2 but not mGlu3 receptors are primarily expressed in CINs [23,31,32], (2) an mGlu2 agonist reduced electrically-induced acetylcholine release from striatal slices [32], similar to a group II agonist that inhibited potassium chloride-induced acetylcholine release from striatal synaptosomes [74], and (3) LY395756, an mGlu2 agonist and mGlu3 antagonist, inhibited striatal dopamine release evoked by optogenetic activation of the thalamostriatal pathway [67]. In addition, an increase in dopamine release in the NAc induced by an mGlu2/3 antagonist [68,75] supports the existence of a basal glutamatergic tone on group II receptors for inhibiting tonic dopamine release.

## 4. Psychostimulants

Extensive pharmacological studies have implicated group II mGlu receptors in the addictive properties of psychostimulants, such as cocaine, amphetamine, and methamphetamine [11,76-83], and in the cue-triggered reward-seeking behavior [84,85]. Since increasing evidence indicates that mGlu2 and mGlu3 receptors are different in their distributions and physiology in the basal ganglia, attention has shifted to focus on the specific role of either subtype. As a prominent presynaptic receptor in the limbic reward circuit, mGlu2 receptors inhibit phasic glutamate and dopamine release in the striatum (see above). As such, mGlu2 receptors are reasoned to suppress neurochemical and behavioral responses to stimulants. In fact, acute cocaine or amphetamine is well characterized to elevate dopamine and glutamate release in the striatum, resulting in hyperlocomotor behavior [86,87]. mGlu2 PAMs (LY487379, TASP0433864, and others), similar to mGlu2/3 orthosteric agonists, reduced locomotor activities induced by acute stimulants (amphetamine or methamphetamine) in rats and mice [88-90], and LY487379 attenuated the acute cocaine-induced activation of the ERK1/2 pathway in the mouse striatum [91]. LY541850, an mGlu2 agonist and mGlu3 antagonist, also reduced hyperlocomotion in acute amphetamine-treated mice [92]. In mGlu2^(−/−)^ mice, acute cocaine administration induced a more rapid and greater increase in glutamate and dopamine release in the NAc, respectively [93]. These results together implicate the mGlu2 subtype in the negative regulation of stimulant actions. Moreover, mGlu2 rather than mGlu3 receptors are central for processing stimulant effects, given that (1) an mGlu2/3 agonist reversed acute amphetamine-stimulated hyperlocomotion in wild-type and mGlu3^(−/−)^ but not mGlu2^(−/−)^ mice [94,95] and (2) the antipsychotic-like effect of an mGlu2/3 agonist on amphetamine-evoked motor responses was absent in mGlu2-lacking Han Wistar rats but not in control Wistar rats [96].

mGlu2 knockout mice exhibited an increase in locomotor sensitization and conditioned place preference in response to repeated cocaine administration, implying the mGlu2-dependent inhibition of the addictive and reinforcing effects of cocaine [93], although a nonsense mutation at the *mGlu2* gene decreased mGlu2 receptor expression and reduced sensitivity to cocaine reward in rats [97]. In an operant self-administration model closely mimicking the addiction condition in humans [98], novel mGlu2 PAMs were used to examine the role of mGlu2 receptors in different aspects of stimulant dependence. Acute systemic administration of mGlu2 PAMs inhibited the reinforcing property of cocaine by reducing cocaine (reinforcer) self-administration in rats [99,100]. The mGlu2 PAMs (BINA and AZD8529) also reduced cue-primed reinstatement of cocaine and methamphetamine self-administration (i.e., relapse) [99,101]. Since these two PAMs did not affect food-seeking behavior, in contrast to the mGlu2/3 agonist LY379268 that reduced motivation for a natural reinforcer, their action to prevent relapse was less likely due to motor deficits or off-target side effects. In an optogenetic study, mice were able to acquire operant self-stimulation of thalamostriatal terminals, indicating a reinforcing nature of stimulation of the thalamostriatal pathway [102]. Notably, this reinforcing property of thalamostriatal activity was reduced by an mGlu2 PAM. Besides, mGlu3^(−/−)^ mice exhibited normal cocaine self-administration, extinction, and reinstatement [103]. The results together support that stimulation of mGlu2 receptors is necessary and sufficient to attenuate the reinforcement and reinstatement of stimulants.

It is worth mentioning that mGlu2 PAMs bind to an allosteric site of mGlu2 receptors that is topographically different from the orthosteric site bound by the endogenous ligand and that PAMs exert their modulatory effects on the receptor only in the presence of glutamate [11,12]. Since allosteric sites are less evolutionarily conserved than orthosteric sites, allosteric modulators may have the potential to gain greater selectivity for individual mGlu subtypes than orthosteric ligands. Moreover, by preserving the temporal aspects of native receptor signaling, mGlu2 PAMs produce less tolerance than exogenous orthosteric agonists. This is of advantage, considering that tolerance following repeated administration of dual mGlu2/3 orthosteric agonists reduces the efficacy of these agonists as addiction medications [11,12].

Expression and function of mGlu2 receptors in the basal ganglia may undergo adaptive changes in response to stimulant exposure, which contributes to the remodeling of excitatory transmission critical for enduring drug-seeking behavior [104]. A variety of anatomical and functional approaches targeting both mGlu2/3 receptors have been utilized to assess the effects of drugs on expression and/or activity of mGlu2/3 receptors and have yielded somewhat varying results [81,105-109]. Multiple studies reported downregulation of the expression and function of mGlu2/3 receptors in the PFC and striatum after repeated investigator-administration of cocaine in a sensitization model or self-administration of cocaine [105-108]. Similarly, methamphetamine self-administration decreased total and surface mGlu2/3 protein levels in the rat dorsal striatum and NAc [110]. Notably, this decrease was reversed by extinction training of methamphetamine self-administration. Using antibodies selective for either the mGlu2 or mGlu3 subtype, a recent study revealed that cocaine self-administration reduced total and surface expression of mGlu2 but not mGlu3 receptors in the NAc core of both male and female rats [111]. This reduction may constitute a key element in a series of glutamatergic adaptations to stimulants. In detail, a well-characterized neuroadaptation model [10] includes an increase in evoked synaptic glutamate release in the NAc during cocaine- and cue-primed reinstatement of cocaine seeking [112-114] coupled with a decrease in basal extrasynaptic glutamate levels after cocaine self-administration [112,115]. The reduction of mGlu2-mediated feedback inhibition of synaptic glutamate release could then serve as a molecular mechanism contributing to an increase in evoked synaptic glutamate release, leading to the reinstatement of cocaine-seeking behavior. As such, restoring mGlu2 expression could reduce this reinstatement. Indeed, ceftriaxone, a *β*-lactam antibiotic, restored mGlu2 expression in the NAc core [111], which likely acted to reduce an increase in synaptic glutamate release [114,116] and prevent reinstatement of cocaine seeking [111]. Similarly, stimulation of remaining mGlu2 receptors with mGlu2 PAMs was sufficient to attain relapse prevention (see above), despite a reduced level of mGlu2 expression after cocaine self-administration.

Recent studies further analyzed the relationship between group II mGlu receptors and methamphetamine. Repeated methamphetamine administration elevated mGlu2/3 expression in the mouse PFC, and activation of presynaptic mGlu2/3 receptors did not inhibit but rather augmented the depolarization-induced D-aspartate release in PFC synaptosomes prepared from methamphetamine-treated mice [117]. These changes in the expression and function of PFC mGlu2/3 receptors may contribute to the remodeling of local excitatory synaptic transmission and thus to the methamphetamine-induced memory deficit. Similarly, methamphetamine-stimulated locomotion and dopamine release in striatal slices were reduced in mGlu2^(−/−)^, but not in mGlu3^(−/−)^, mice [118]. Future studies need to define the accurate roles of mGlu2 versus mGlu3 receptors in processing methamphetamine action under different experimental conditions (types of drugs, species, models, dosing, timing, brain regions, etc.).

## 5. Alcohol

Alcohol abuse is a leading health problem worldwide with limited effectiveness of pharmacotherapies. Available evidence supports a link between limbic mGlu2 receptors and alcoholism [119-121]. The mGlu2 PAM AZD8529 modestly reduced alcohol self-administration at doses that did not affect operant responses to a non-drug reinforcer, saccharin, in rats [122]. AZD8529 also blocked cue-induced reinstatement of alcohol seeking [122], although the mGlu2 PAM BINA had no effect [123]. Additionally, the mGlu2 PAM LY487379 reduced alcohol relapse in both male and female rats [124]. Thus, activation of mGlu2 likely attenuates the reinforcing value of alcohol and relapse-like behavior.

It was reported that ‘optimistic’ rats showed lower alcohol consumption than ‘pessimistic’ rats [125]. A possible mechanism for this may involve an elevated level of mGlu2 receptor expression in the amygdala of ‘optimistic’ rats. On the other hand, loss of mGlu2 may enhance the vulnerability to alcoholism. Alcohol-preferring (P) rats are an animal model that mimics many important aspects of human alcoholism, such as tolerance, physical dependence, alcohol-seeking behavior, and tendency to relapse following a period of abstinence [126,127]. In these P rats, a naturally occurring stop codon mutation at cysteine 407 in *Grm2* (cys407*) was recently identified, which leads to the loss of functional mGlu2 protein expression [64,128]. This *Grm2* mutation was linked to increased alcohol consumption and preference. Such linkage in P rats was substantiated by elevated alcohol consumption and preference in *Grm2*^(−/−)^ mice. Moreover, an mGlu2 PAM lost its ability to block alcohol relapse in P rats [122]. An mGlu2/3 antagonist escalated alcohol self-administration in Wistar rats and alcohol-non-preferring rats that express functional mGlu2 receptors [64]. Of note, the cys407* mutation is common in some commercially available rats [128]. Therefore, caution needs to be exercised when selecting strains and sources of rats for neurochemical and behavior studies involving mGlu2 receptors.

Cre-dependent and neuron-specific knockdown of mGlu2 receptors in the infralimbic cortex was sufficient to generate a phenotype of excessive alcohol seeking in nondependent rats [129]. However, short-hairpin RNA-mediated knockdown of mGlu2 receptors in the rat prelimbic cortex by a magnitude of ~40% did not alter voluntary alcohol drinking [130]. Future studies will need to examine the effect of mGlu2 knockdown on alcohol-seeking behavior at a range of different percentages or in additional limbic reward sites.

Chronic alcohol exposure produces maladaptive changes in mGlu2 activity. Human anterior cingulate cortex from patients with chronic alcohol exposure showed a reduced level of mGlu2 transcripts [131]. Chronic alcohol exposure reduced mGlu2 but not mGlu3 mRNA expression in the rat PFC [131]. The mGlu2 reduction occurred specifically in infralimbic-accumbal glutamatergic projection neurons. Similarly, ethanol-dependent mice showed a lowered level of mGlu2 proteins in the NAc core [132]. Functionally, the mGlu2 autoreceptor activity in inhibiting glutamate release was downregulated at the corticoaccumbal synapses in the NAc [100]. A similar downregulation was seen in the prelimbic cortex, although not the NAc core, of rats that developed alcohol use disorder [133]. In another study, mGlu2-LTD at corticostriatal/thalamostriatal synapses in the dorsolateral striatum was impaired after chronic ethanol exposure in adolescent mice [134]. An mGlu2 PAM fully rescued mGlu2-LTD in ethanol-treated mice. Acute ethanol exposure to striatal slices failed to disrupt mGlu2-LTD. Since mGlu2 mRNA expression in several cortical regions and the thalamus and mGlu2 protein expression in the dorsal striatum were not significantly altered by ethanol, the downregulation of mGlu2 function in inducing LTD may be mediated by other mechanisms [134]. Together, these data imply a downregulation of mGlu2 autoreceptor function in the human and rodent striatum or in other limbic regions as a critical neuroadaptation component and a key mediator of alcohol dependence. Normalization of mGlu2 function could then prevent alcohol reinstatement. Indeed, restoration of mGlu2 expression in the infralimbic neurons projecting to the NAc via viral-mediated gene transfer attenuated excessive cue-induced alcohol seeking [131]. Finally, intermittent access to ethanol induced a cell type-specific increase in synaptic strength and mGlu2/3 receptor plasticity on mouse PFC intratelencephalic pyramidal cells, providing an additional rationale for developing mGlu2 and/or mGlu3 selective agents for treating alcohol use disorders [135].

## 6. Nicotine and Opioids

Nicotine is another addictive substance associated with mGlu2 participation. Given the critical involvement of hyperactive glutamatergic transmission in nicotine dependence [136], compounds that reduce glutamatergic transmission have therapeutic potential. In fact, systemic administration of the mGlu2/3 receptor agonist LY379268 and injection of this agonist into the VTA or NAc decreased nicotine, but not food, self-administration in rats [137]. Similar to the mGlu2/3 agonist, mGlu2 selective PAMs decreased nicotine self-administration in rats [138,139] and squirrel monkeys [140]. Both LY379268 and the mGlu2 PAM AZD8529 reduced a nicotine-induced increase in dopamine release in the rat NAc [140,141], and LY379268 notably exerted this effect only in the presence of a nicotine-associated context [141]. Thus, mGlu2 receptors negatively regulate the reinforcing property of nicotine consumption, and inhibition of NAc dopamine release contributes in part to this event. Additionally, mGlu2 PAMs blocked cue-primed reinstatement of nicotine seeking in rats [139] and squirrel monkeys [140]. These findings link mGlu2 activity to nicotine relapse and support the therapeutic value of mGlu2 PAMs for relapse prevention [13,142]. Of note, adolescent nicotine exposure reduced mGlu2 protein levels and function on presynaptic glutamatergic terminals in the rat PFC [143]. Restoring mGlu2 receptor activity in the local PFC rescued cognitive impairments.

As with drugs discussed above, the dual mGlu2/3 agonists inhibited rewarding and reinstatement of morphine or heroin seeking [144-148]. In addition, the mGlu2 PAM ADX106772 reduced seeking behavior in male Wistar rats treated with oxycodone (the most abused prescription opioid) [149]. A study with transgenic mGlu2 knockout rats found that deletion of mGlu2 receptors profoundly altered multiple addictive properties of opioids, including increased NAc dopamine release in response to acute heroin, enhanced behavioral sensitization to repeated heroin, escalated heroin self-administration, and more potent analgesic effect with morphine administration [150]. These results suggest that a lack of mGlu2 receptors is a risk factor for opioid abuse and that a low level of mGlu2 expression may present a useful biomarker for assessing vulnerability to opioid addiction. In addition, mGlu2/3 protein expression and mGlu2/3-LTD at corticoaccumbal synapses in the NAc were downregulated after repeated morphine administration [151,152]. Future studies will assess changes and roles of individual mGlu2 or mGlu3 subtypes in opioid abuse.

## 7. Conclusions

G*α*_i/o_-coupled mGlu2 receptors reside on glutamatergic nerve terminals and serve as presynaptic autoreceptors to produce feedback inhibition of glutamate release. A high level of presynaptic mGlu2 receptors is distributed in the basal ganglia, especially in the CPu and NAc. By modulating glutamatergic transmission and synaptic plasticity in the striatum, mGlu2 receptors participate in controlling motivational, reward, motor, and cognitive functions. Chronic exposure to addictive drugs such as psychostimulants (cocaine and amphetamines), nicotine, and alcohol reduces the expression and function of mGlu2 autoreceptors in the striatum. This impairs the mGlu2-mediated feedback inhibition of glutamate release, reshaping synaptic glutamatergic transmission and plasticity critical for enduring drug-seeking behavior. As a result, restoring mGlu2 activity attenuates drug taking and seeking. Increasing preclinical evidence supports the potential of mGlu2 subtype-selective agents (orthosteric agonists and PAMs) as pharmacotherapies for treating addiction.

While a great deal of progress has been made in the understanding of mGlu2 receptor biology and its roles in drug addiction, detailed molecular mechanisms are incompletely understood. Future studies are warranted to elucidate the underlying mechanisms for the regulation of mGlu2 receptors and their contributions to drug seeking. Anatomically, precise localizations of mGlu2 receptors on glutamatergic nerve terminals that project to the striatum from different source regions (cortex, thalamus, and others) and make excitatory asymmetric synapses with distinct subsets of projection (striatonigral versus striatopallidal) neurons or interneurons need to be mapped at the ultrastructural level. Physiologically, molecular mechanisms underlying the basal and activity-dependent regulation of mGlu2 receptors are unclear. Future studies will investigate the role of posttranslational modifications, such as phosphorylation, palmitoylation, ubiquitination, etc., in the dynamic regulation of the receptor. Finally, the mGlu2 receptor in its adaptations to drug exposure and its roles in mediating drug effects needs to be compared with other mGlu subtypes, especially the mGlu3 subtype. It is likely that multiple mGlu subtypes, as well as ionotropic glutamate receptors, work in concert to reshape limbic glutamatergic transmission to a state of pro-addiction.

## Abbreviations

cAMP: cyclic adenosine monophosphate
CIN: cholinergic interneuron
CPu: caudate putamen
ERK: extracellular signal-regulated kinases
GABA: *γ*-aminobutyric acid
GIRK: G-protein-coupled inwardly rectifying K^+^
LTD: long-term depression
mGlu: metabotropic glutamate
MSN: medium spiny projection neurons
NAc: nucleus accumbens
PAM: positive allosteric modulator
PFC: prefrontal cortex
PKA: protein kinase A
SNr: substantia nigra pars reticulata
SNc: substantia nigra pars compacta
VGCC: voltage-gated Ca^2+^ channels
VTA: ventral tegmental area
